# Involvement of Protease-Activated Receptor2 Pleckstrin Homology Binding Domain in Ovarian Cancer: Expression in Fallopian Tubes and Drug Design

**DOI:** 10.3390/biomedicines12010246

**Published:** 2024-01-22

**Authors:** Jeetendra Kumar Nag, Sorina Grisaru-Granovsky, Shunit Armon, Tatyana Rudina, Priyanga Appasamy, Rachel Bar-Shavit

**Affiliations:** 1 Sharett Institute of Oncology, Hadassah-Hebrew University Medical Center, Jerusalem 91120, Israel; nagj3@ccf.org (J.K.N.); rutanya0@gmail.com (T.R.); priyanga.appasamy@mail.huji.ac.il (P.A.); 2 Department of Obstetrics and Gynecology, Shaare-Zedek Medical Center (SZMC), Hebrew-University, Jerusalem 9103102, Israel; granovs@012.net.il (S.G.-G.); shunita@szmc.org.il (S.A.)

**Keywords:** G-protein coupled receptors (GPCRs), protease-activated receptors (PARs), fallopian tubes (FTs), pleckstrin homology (PH) domain, ovarian cancer

## Abstract

Studying primordial events in cancer is pivotal for identifying predictive molecular indicators and for targeted intervention. While the involvement of G-protein-coupled receptors (GPCRs) in cancer is growing, GPCR-based therapies are yet rare. Here, we demonstrate the overexpression of protease-activated receptor 2 (PAR2), a GPCR member in the fallopian tubes (FTs) of high-risk BRCA carriers as compared to null in healthy tissues of FT. FTs, the origin of ovarian cancer, are known to express genes of serous tubal intraepithelial carcinoma (STICs), a precursor lesion of high-grade serous carcinoma (HGSC). PAR2 expression in FTs may serve as an early prediction sensor for ovarian cancer. We show now that knocking down Par2 inhibits ovarian cancer peritoneal dissemination in vivo, pointing to the central role of PAR2. Previously we identified pleckstrin homology (PH) binding domains within PAR1,2&4 as critical sites for cancer-growth. These motifs associate with PH-signal proteins via launching a discrete signaling network in cancer. Subsequently, we selected a compound from a library of backbone cyclic peptides generated toward the PAR PH binding motif, namely the lead compound, P*c*(4-4). P*c*(4-4) binds to the PAR PH binding domain and blocks the association of PH-signal proteins, such as Akt or Etk/Bmx with PAR2. It attenuates PAR2 oncogenic activity. The potent inhibitory function of P*c*(4-4) is demonstrated via inhibition of ovarian cancer peritoneal spread in mice. While the detection of PAR2 may serve as a predictor for ovarian cancer, the novel P*c*(4-4) compound may serve as a powerful medicament in STICs and ovarian cancer. This is the first demonstration of the involvement of PAR PH binding motif signaling in ovarian cancer and P*c*(4-4) as a potential therapy treatment.

## 1. Introduction

Illuminating primordial events and elucidating their molecular pathogenesis that leads to cancer is pivotal for identifying predictive molecular indicators and for appropriate targeted interventions. Notably, only a minority of cases of ovarian cancer are attributed to known genetic disorders [1,2], and mostly this cancer does not occur due to a hereditary background. Ovarian carcinoma differs from other solid cancers that metastasize by lymphatic or hematogenous paths, and instead spreads by disseminating primarily within the peritoneal cavity, anchoring to the mesothelium, and leading to omental, diaphragm, and bowel serosa metastasis [3,4]. The high death rate in ovarian cancer is largely due to peritoneal spread at the time of diagnosis and the often-severe drug resistance to current treatment [5]. In spite of years of extensive research, the major complication in effective ovarian cancer management is due to lack of information on the mechanistic underpinnings that control tumor metastasis [6,7]. In order to advance treatment plans, it is essential to better comprehend the origin of ovarian cancer and to elucidate the molecular signaling pathways involved.

Although the contribution of G-protein-coupled receptors (GPCRs) in cancer malignancy is growing, GPCR-centered therapies are rare [8,9]. It is well acknowledged that within the dynamically active microenvironment of a tumor, matrix-bound as well as soluble proteases are engaged in maintaining tumor growth and progression. For instance, there is a continuous cross-talk between the family of mammalian protease-activated receptors (PARs) and proteases in the tumor microenvironment. PARs, a subgroup of GPCRs, is comprised of four family members (PAR1-4), all of which are uniquely activated via proteolytic cleavage at their N-terminal extracellular portion. We have identified motifs within the C-tails of PAR1,2&4 which are critical for cancer growth and development [10,11]. These motifs are the binding sites of pleckstrin homology (PH) domain, present in various signal proteins. It triggers a spectrum of intermolecular interactions to launch a discrete signaling network in cancer [12]. Accordingly, we generated a library of backbone cyclic peptides directed toward the PAR PH-bonding motif, out of which a lead compound termed P*c*(4-4), of “drug-like” properties was selected [11].

The ongoing challenge associated with cancer research is the designation of an appropriate patient population that will best respond to personalized therapy medication(s). The aberrant β-catenin signaling pathway driven by Wnt/frizzled (FZD) receptors is frequently observed in cancer. FZDs are negatively controlled by RNF43, an E3 ubiquitin ligase that potently degrades FZD levels [13,14]. On the other hand, inactivating RNF43 mutations, found in several types of cancer including cancers of the gastrointestinal (GI) system, endometrium, and in ovarian cancer [15], lead to high β-catenin levels and increased FZD levels [16]. As a result, these patients are dependent on high FZD functional consequences. RNF43 mutations provide a unique opportunity to identify a best responding patient population to upstream Wnt/FZD inhibitors (avoiding a high mutation level downstream of Wnt/β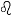-catenin signaling). In this context, we recently described RNF43 as a novel negative regulator of PAR2, capable of inhibiting cell surface PAR2 expression and the PAR-induced β-catenin stabilization pathway [17]. As a result, PAR2 joins FZDs as a target for RNF43 negative regulation.

Evidence from genetically engineered mouse models along with human pathological tissue samples led to the current paradigm that the origin of high-grade serous carcinoma (HGSC), the most frequent type of ovarian cancer, resides within fallopian tubes (FTs). Lesions occurring primarily in the FTs develop further to metastasize to the nearby ovaries, peritoneum, and lymph nodes [18,19]. Pathological samples found in serous tubal intraepithelial carcinomas (STIC), a precursor lesion of HGSC, display a “p53 mutation” signature, typical of HGSC, and which occurs in all precursor lesions [19,20,21]. However, it seems that p53 missense or nonsense mutations by themselves are insufficient to lead to an ovarian cancerous transformation. Indeed, most p53 mutant cells do not divide and may remain indolent. It is most likely that in addition to the requisite of archetypal p53 signature [22], other genes are required to progress toward malignancy [23,24,25]. While the above examples are circumstantial, a direct proof-of-concept came from elegant studies by Perets et al. [26] generating a genetically engineered mouse model of HGSC. It utilizes the PAX8 transcription factor, which is vital for the female genital tract excluding the ovaries, as a bait. The model recapitulates all stages in FTs during the progress to HGSC, demonstrating its primary involvement in the process.

In the present communication, we asked whether the PAR2 stem cell marker is part of an ancestral gene landscape responsible for HGSC. In addition, we addressed the molecular mechanism of PAR2-induced ovarian cancer which can serve as a platform for the design of a therapy strategy. For this, we utilized bioinformatic analyses, protein immunoprecipitation, immunohistochemistry (IHC), RT-PCR, Matrigel invasion in vitro, and xenograft mouse models in vivo. We demonstrate the overexpression of PAR2 in FTs of high-risk BRCA carriers as compared to null expression in healthy tissues of FT. Based on the notion that FTs STICs are the origin of HGSC; identification of PAR2 in FTs for early detection is important. As part of elucidating the molecular mechanism of PAR2-induced ovarian cancer we now demonstrate the powerful role of PAR PH binding motif(s) as a potent platform for powerful drug design. While the contribution of PAR2 in ovarian cancer has been previously shown [27,28,29], we now present evidence demonstrating the prevailing role of P*c*(4-4) directed toward PAR2-PH binding domain as a therapy treatment in ovarian cancer. P*c*(4-4) effectively inhibits PAR2-PH-Akt association and ovarian cancer peritoneal dissemination in a mouse model in vivo. Overall, it is concluded that PAR2 expression in FTs can serve as an early predictor of ovarian cancer. It is suggested that this may represent an ovarian cancer driver gene and a platform poised for tailored made therapy.

## 2. Materials and Methods

### 2.1. Animal Models

The mice used in the study were handled according to the rules of the Hebrew University Ethics Committee (AAALAC standard). Mice (HSD: athymic female nude-Foxn1Nu Nu/Nu mice) were retained under specific pathogen-free (SPF) settings at the Hadassah Medical Center animal facility unit of the Hebrew University and were frequently screened for standard pathogens. The animal ethics number is MD-20-15924-5.

### 2.2. Human Tissues

In accordance with the Ethical Committee approval conditions (SZMC-0433-21) and the present study group’s criteria (low-risk healthy women, non-BRCA carriers, high-risk BRCA carriers who experienced risk-reducing surgery, and ovarian cancer patients), the paraffin-embedded fallopian tubes samples were retrieved from the Department of Pathology. The primary formal H&E stained slides were assessed by Dr. Shunit Armon (Department of Obstetrics and Gynecology, Shaare-Zedek Medical Center, Jerusalem 9103102, Israel).

### 2.3. Patient Data Analysis

Patient data analysis was carried out using web search analyses of the Gene Expression Profiling Interactive Analysis (GEPIA) server. A group of 426 ovarian cancer patients was analyzed and compared with samples from 88 healthy controls with normal ovarian tissue.

Fallopian tube epithelium was removed by surgery from normal healthy women and women carriers of BRCA1/2 mutations. The samples were obtained from women aged 43–75 years. The samples were obtained from women with BRCA 1/2 mutations who underwent prophylactic risk-reducing salpingo-oophorectomy and women who underwent fallopian tube removal for benign gynecological conditions with no family history of ovarian or breast cancer; none were pregnant or using hormone replacement therapy (considered as normal FT tissues). The BRCA set included women with BRCA1 mutations and women with BRCA2 mutations. The BRCA mutation was evaluated by standard genetic analysis covered by the Israeli National Health Insurance genetic laboratories or the licensed SZMC Human Genetics Department. The selection of the representative paraffin-embedded blocks and the preparation of the histologic slides was performed by the staff of the Pathology Dept and one of the authors (SA). The Institutional Ethical Approval (SZMC-0433-21) was obtained and waived from written consent on the basis that the study samples were selected based on the Israeli ID and recorded diagnosis and de-identified for the research team.

### 2.4. Cell Culture

ES2 [30], OVCAR8, OVCAR3, and HEK293 (American Type Culture Collection (ATCC), Manassas, VA, USA) were maintained in DMEM, supplemented with streptomycin, 50 U/mL, 1 mM l-glutamine, 50 µg/mL, penicillin (GIBCO-BRL, Gaithersburg, MD, USA), and 10% fetal calf serum (Biological Industries, Migdal HaEmek, Israel). Cells were retained in a humidified incubator with 8% CO_2_ at 37 °C. Briefly, ES2 ovarian cancer cells are originated from a primary clear cell carcinoma of the ovary presenting in a 47-year-old African American patient. OVCAR-3(NIH OVCAR3 ATCC HTB-161) are epithelial cells, isolated in 1982 from the malignant ascites of a patient with progressive adenocarcinoma of the ovary. OVCAR8 is a high-grade serous ovarian cancer. Lineage subtype is ovarian epithelial tumor. Lineage sub-subtype is of high-grade serous ovarian cancer. Molecular subtype is of high-grade serous ovarian cancer.

### 2.5. Cell transfections, Plasmids, and PAR Activation

Cell monolayers of 70–80% confluency were transfected using poly(ethyleneimine) (PEI) transfection reagent (Polysciences, Warrington, PA, USA) with 0.5–1 μg of plasmid DNA following the manufacturer’s instructions. After 48 h, transfection cells were collected, and RNA or protein lysates were prepared. Human PAR2 (hPar2/f2rl1) plasmid was provided by Dr. Morley D. Hollenberg, Faculty of Medicine, University of Calgary, Calgary, AB, Canada. pCMV-dR8.2 dvpr (cat #8455) and pCMV-VSV-G (cat #8454) plasmids were obtained from Addgene. Activation of PAR2 was carried out by application of 200 µM of the synthetic peptide “SLIGKV”.

### 2.6. Small Hairpin (sh) RNA Preparation and Generation of Viral Particles

The desired sequence of sh-RNA of Par2 as outlined below was cloned between XhoI and HpaI restriction sites of the GFP-plentilox3.7 (pLL3.7) lentiviral vector following the protocol provided by the Addgene website (Cat# 11795). The target sequences were as follows: sh-Par2i 5′-GGAAGAAGCCTTATTGGTA-3′ and sh-Par2ii:5′-GCT CTTTG TAATG TGCTTA-3. In order to generate the viral particles, three plasmid systems that include packaging (CMVD R8.91), envelope (CMV-VSV-G), and sh-Par2-pLL3.7 vector were transfected into HEK293 cells by PEI. Fresh medium was added after 24 h. The medium was collected on day 3 after transfection. A 10-fold concentration of the viral particles was carried out following centrifuging at 40,000 rpm for 2 h.

### 2.7. Real-Time PCR (qRT PCR) and RT PCR

A GenElute RNA kit (Sigma-Aldrich, Burlington, MA, USA) was used for RNA extraction from cells. A total of 1 µg of RNA was reverse transcribed using reverse transcriptase (Promega, Madison, WI, USA) in order to prepare cDNA. Specific forward and reverse primers were used for qRT-PCR analysis for each gene studied. The 6ng cDNA templates were used with 500 nM gene-specific primers in qRT-PCR, with triplicates using 2× PerfeCTa SYBR Green mix (Agentek, Tel Aviv-Jaffa, Israel) on an automated rotor gene system RG-3000A (Corbett research, Sydney, Australia). Table 1 includes the list of primers. Three independent qRT-PCR experiments for all the data obtained were analyzed using the 2-∆∆Ct method according to the manufacturer’s instructions, and results were expressed as fold change over the indicated controls.

The following primer set was used.

**Table 1:** List of primers used:

LGR5	F: 5′ CCAACCTCAGCGTCTTCACC 3′ R: 5′ GGAGACTGGGCAGGGGATT 3′
CD44	F: 5′ CTGGGGACTCTGCCTCGT 3′ R: 5′ ACGTGGAATACACCTGCAAAGC 3′
ALDH1	F: 5′ CACGCCAGACTTACCTGTCC 3′ R: 5′ TGCCACTCACTGAATCATGCC 3′
Oct-4	F: 5′-GAG AATTT GTT CCT GCA GTG C-3′ R: 5′-GTT CCC AAT TCC TTC CTT AGT G-3′
CD166	F: 5′ GATCTCCGCCACCGTCTTC 3′ R: 5′ CGTCAAGTCGGCAAGGTATGG 3′
Par2	F: 5′-GGC CAA TCT GGC CTT GGC TGA C-3′ R: 5′-GGC AGG AAT GAA GAT GGT CTG-3′
Par4	F: 5′ CCCAGCGTCTACGACGAGA 3′ R: 5′ CACAGACTTGGCCTGGGTAG 3′
GAPD	F: 5′-CCA CCC ATG GCA AAT TCC ATG GCA-3 R: 5′-TCT AGA CGG CAG GTC AGG TCC ACC-3′

### 2.8. Preparation of Cell Lysates, Immunoprecipitation, and Western Blot

Immunoprecipitation (IP) of cell lysate proteins was carried out following solubilization in CelLytic™ M buffer (Sigma-Aldrich, St. Louis, Mo, USA). It routinely contained 10 mM Tris-HCl (pH7.4), 1 mM EDTA, 150 mM NaCl, and 1% Triton X-100. All lysis buffers were complemented with 1 mM phenylmethyl sulfonyl fluoride (PMSF), a protease inhibitor cocktail, and 1 mM Na-orthovanadate (Sigma, St. Louis, MO, USA) to avoid protein degradation. For IP, cell lysates were incubated for 20 min at 4 °C and then sonicated. Finally, collection of soluble supernatant was performed after centrifugation for 20 min at 4 °C at 12,000 rpm. Cell lysates were separated on 8–12% SDS-PAGE followed by transfer to an Immobilon-P membrane (Millipore, Bedford, MA, USA). Blocking of the membranes was carried out, followed by probing with the suitable primary antibodies. The anti-PAR2 (AB180953; Abcam, Waltham, MA, USA: SC-13504 Santa Cruz) and anti-AKT (AB8805; Abcam, Waltham, MA, USA) antibodies were suspended in 3% BSA in 10 mM Tris–HCl pH 7.5, 100 mM NaCl, and 0.1% Tween-20. After extensive washing, incubation of the blots with secondary antibodies conjugated to horseradish peroxidase was performed. Enhanced chemiluminescence (ECL) reagent (Pierce, Rockford, IL, USA) was applied for the detection of immunoreactive bands. Proteins of cell lysates (400–800 μg) were used for IP analysis. Anti-PAR2 antibodies (SC-13504; Santa Cruz) were added to the cell lysates and processed as described above. Anti-Etk/Bmx (BD Transduction Labs, SD, USA), or anti-Akt (Cell Signaling Technology) antibodies were applied on Western blots for the detection of PAR2 immunoprecipitants. Anti-β-actin served as a control housekeeping gene (A5441; Sigma-Aldrich, St. Louis, MO, USA).

Anti-green fluorescent protein (GFP) abs (Sigma, St. Louis, MO, USA) were applied on a cell monolayer for the detection of peptide cell penetration.

### 2.9. Matrigel Invasion Assay

Chemotaxis blind-well chambers of 13 mm width filters were utilized. Filters of 8 mm pore size (Costar Scientific Co., Washington, DC, USA), with polyvinylpyrrolidone-free polycarbonate, were covered with basement membrane Matrigel (50 μL of 1 mg/mL). A layer of Matrigel (50 mg/filter) was smeared as described previously [31]. Matrigel was diluted with cold distilled water to the desired final concentration prior to application on the filters, and then the filters were dried under a hood. ES2 cells (1.8 × 10^5^), were added to the upper chamber and suspended in DMEM containing 0.1% bovine serum albumin (BSA). Then, 3T3 fibroblast-conditioned medium was applied to the lower compartment of the Boyden chambers as a chemoattractant. Next, SLIGKV was added to the cells. Non-SLIGKV-activated cells were used as a control. The chambers were incubated under a humidified incubator with 8% CO_2_ at 37 °C overnight. At the end of this time, cells on the upper filter were carefully removed using a cotton swab. Then, the filters were fixed and stained with hematoxylin and eosin (Sigma-Aldrich, St. Louis, MO, USA). Filter invading cells were counted from different areas of the filter underneath.

### 2.10. Peptide Synthesis

Peptide PAR2-acetylated (Ac) and amidated (Am), peptide PAR2 4Tfmoc, peptide PAR2 6Tfmoc, and peptide PAR2 8Tfmoc were synthesized by Prof Chaim Gilon (Hebrew University Dept of Chemistry, Jerusalem Israel as outlined [11]). All peptides are soluble in DDW, highly stable, and are of <96% HPLC purity.

### 2.11. Pc(4-4) Cyclic Peptide

The detailed design, synthesis, and characterization of PAR2-PH binding domain peptides and the P*c*(4-4) compound (synthesized by Prof Chaim Gilon, Hebrew University Dept of Chemistry, Jerusalem Israel) was carried out as described at length earlier [11].

### 2.12. Tumor Xenograft Mouse Model

The model for ovarian cancer was employed following intraperitoneal (i.p.) inoculation of ovarian cancer cells. Briefly, wt ES2 cells and sh-Par2-ES2 were starved overnight, and they were then treated the next day with SLIGKV (200 µM each) for 4 h prior to mice inoculation. After adequate washing, i.p. injection of cells (1 × 10^6^) was performed on six- to eight-week-old female Hsd: athymic Nude mice purchased from Harlan (Harlan Holding, Harlan Laboratories Israel, and HBI Biotech Sciences) (nude mice; n = 7). As part of the experiment, ES2 cells were treated with P*c*(4-4) (5 mg/kg) by i.p. injection (3× times per week). Mice were monitored every other day, until they developed noticeably swollen abdomens. Mouse body weight was tracked and mice were sacrificed when general health signs were determined to be declining. Mice were killed under aesthetic conditions by cervical dislocation, as specified under the Hebrew University Institutional Animal Committee’s rules (ethics number: MD-20-15924-5) in order to avoid unnecessary suffering.

### 2.13. Immunohistochemistry

FT-derived paraffin-embedded slides were used for immunohistochemistry (IHC) analyses. The slides were incubated with 3% H_2_O_2_ after deparaffinization and rehydration and prior to antigen retrieval. Then, heating for 20 min in a microwave oven for antigen unmasking was carried out with 1× antigen retrieval citrate buffer (Cat# ab93678, Abcam). CAS-Block (Cat# 008120, Invitrogen, Waltham, MA, USA) was applied for blocking, and then the slides were incubated with anti-PAR2 (AB180953; Abcam, Waltham, MA, USA) abs. Peroxidase-conjugated antibody (Abcam, Cambridge, UK) was applied to the slides after washing. DAB substrate kit (Cat# 34002, Thermo Scientific, Waltham, MA, USA), was used for color development followed by counter staining with Mayer’s hematoxylin (Cat# 3801582E, Leica, Wetzlar, Germany). Secondary antibodies only (with no primary antibodies) were used as controls, showing low background staining in all cases.

### 2.14. Statistical Analysis

All experiments were carried out in triplicate, and the data were represented as mean ± standard deviation (SD). The significance of the difference of tested samples in comparison to controls was determined by performing either analysis of variance (ANOVA) with Tukey’s multiple comparison post-test (GraphPad Prism 6.0) or Student’s *t*-test when required. *p* < 0.05 was considered significant; *p* < 0.01 was highly significant; *p* < 0.001 was very highly significant.

## 3. Results

### 3.1. PAR2 Is Highly Expressed in FTs of BRCA Carriers

**Figure 1:**
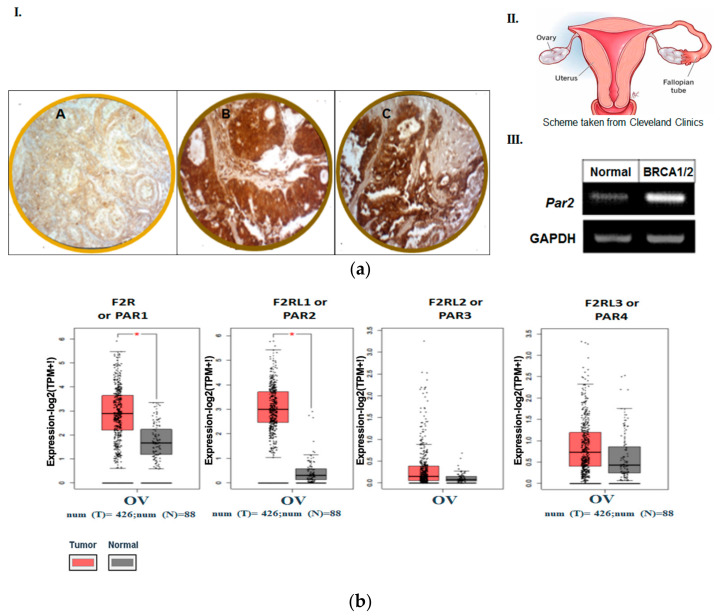
(**a**) Expression of PAR2 in FT BRCA1/2 individuals but not in normal FTs. (**A**): The expression of PAR2 in FT BRCA1/2 individuals but not in normal FTs: IHC. (**I**) Weak to no staining in normal fallopian tube (FT) tissue (**A**) Strong staining of PAR2 (Abcam Ab; 1:100) in FT tissue specimen of BRCA1/2 carrier (**B**,**C**). Magnifications are ×20, scale is 50 μm. A total of 20 individuals were tested (14/20 positive). (**II**) Scheme of FTs and ovaries (taken from Cleveland Clinics). (**III**). RT-PCR analysis of Par2 levels in FT from healthy controls and BRCA1/2 carriers. GAPDH serves as a control housekeeping gene. (**b**) Landscape of PARs in ovarian serous cystadenocarcinoma. The landscape of PARs in ovarian serous cystadenocarcinoma was determined by GEPIA server. Data analyses of PAR family members conducted on a large cohort of ovarian cancer patients.

In order to study the possible role of PAR2/f2rl1 in the etiology of ovarian cancer, we have undertaken the task of evaluating levels of PAR2 expression in FTs of normal and high-risk carriers of BRCA1/2 mutations. Tissue samples were obtained from this high-risk population of women who experienced an ovarian cancer risk-reducing salpingo-oophorectomy. As demonstrated in Figure 1a, an abundant high expression level of PAR2 can be seen in these tissues (14 out of 20 samples studied) compared to no expression in FT tissues obtained from low-risk BRCA non-carrier individuals (18 out of 20 samples). This was demonstrated both by IHC staining and quantitative RT-PCR analyses.

### 3.2. The Pattern of Members of PAR Family in Human Ovarian Tumors

In order to substantiate the central role of PAR family members in the etiology of ovarian cancer, we evaluated their levels using a Gene Expression Profiling Interactive Analysis (GEPIA) server. A cohort of 426 patients with ovarian cancer was analyzed and compared with samples from 88 healthy low-risk controls with normal ovarian tissue. A high expression level of PAR1/f2r; PAR2/f2rl1 and PAR4/f2rl3 was demonstrated in ovarian carcinoma. The highest level was observed in PAR2/f2rl1 in serous adenocarcinoma versus low levels in normal ovarian tissues (Figure 1b). This corresponds with data published by Jiang Yuhong et al. [28] showing high expression levels of PAR2 in ovarian cancer in a large patient population using several web server tools, such as the Cancer Genome Atlas (TCGA), Genotype-Tissue Expression (GTEx), and Gene Expression Omnibus (GEO) databases. This result points to the potentially significant role of PAR2/f2rl1 in the ovarian carcinomas’ etiology.

### 3.3. Knockdown of Par2/f2rl1 Markedly Inhibits Stem Cell Markers In Vitro and Ovarian Cancer Metastasis In Vivo

**Figure 2:**
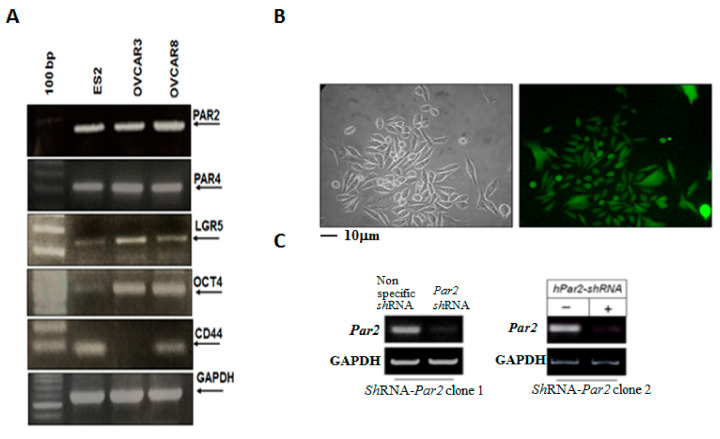
Levels of PAR2 and PAR4 as well as stem cell markers in various ovarian cancer cell lines. Preparation of sh-Par2/f2rl1-GFP. Characterization of knockdown PAR2 ES2 cells. (**A**) RT-PCR analysis of stem cell markers. ES2, OVCAR3, and OVCAR8 ovarian cancer cell lines were evaluated for levels of stem cell markers. GAPDH served as a housekeeping gene. (**B**) ES2 metastatic ovarian cells were infected with a lentivirus containing either Par2-shRNA cassette or scrambled shRNA cassette. GFP fluorescence indicated the infection efficiency. More than 90% of the cells showed marked expression of GFP, indicating effective infection. (**C**) Clones of Par2-shRNA effectively inhibit hPar2 mRNA levels in ES2 cells. GAPDH served as a housekeeping gene.

RT-PCR analyses of several aggressive ovarian cancer cell lines revealed high expression levels of Par2/f2rl1 and Par4/f2rl3 (Figure 2A). The stem cell markers LGR5, Oct4, and CD44 were highly expressed in the ovarian cancer cell lines ES2, OVCAR8, and OVCAR3 (Figure 2A). Next, we moved on and knocked down Par2/f2rl1 in ES2 cells (Figure 2B,C). As can be seen, robust expression of the shRNA lentiviral expression of shRNA-Par2 is obtained following the infection of the Par2-shRNA -GFP cassette observed by the abundant fluorescence of the ES2 cell monolayer (Figure 2B). A marked inhibition of Par2 levels is shown in two clones of shRNA-Par2 as compared with scrambled sh-RNA (Figure 2C).

We then studied the impact of Par2/f2rl1 gene silencing in an ovarian cancer mouse model, in vivo. The shRNA constructs of Par2 were prepared in a lentilox3.7 vector and produced appropriate sh-Par2-silenced viruses. Subsequently, we infected ES2 cells for knockdown of the high Par2/f2rl1 endogenous levels of ES2 cells. As can be seen, GFP-sh-RNA-Par2-infected ES2 cells had an abundant presence of green cells, indicative of sh-Par2 levels (Figure 2B). The knockdown sh-Par2 cells exhibited more epithelial-like morphogenesis versus the ES2 mesenchyme elongated wt cells. Once we had generated Par2-silenced clones (Figure 2C), we injected wt ES2 (1 × 10^6^ cells) and shRNA-Par2/ES2 clones (1 × 10^6^ cells) i.p. into female mice.

**Figure 3:**
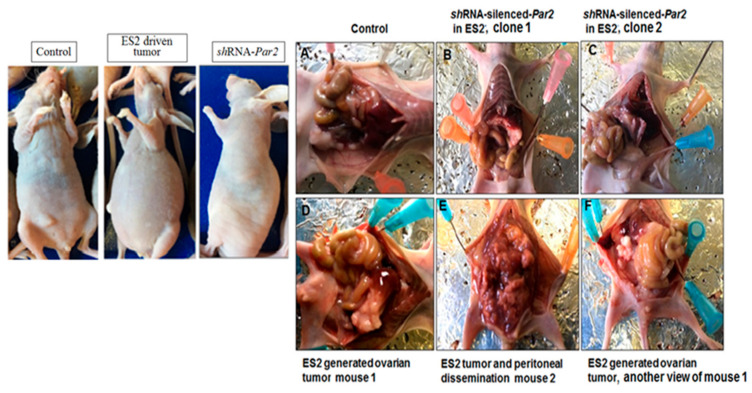
Knockdown of PAR2 inhibits ovarian cancer growth and peritoneal dissemination. Mice were injected i.p. with either wt ovarian cell carcinoma ES2 cells (1 × 10^6^) or with clones of shRNA-hPar2 ES2 cells. Left panel: image of representative intact control, wtES2 and ES2 shRNA-Par2-injected mice. In the presence of ES2 cells (of high PAR2 levels), a swollen peritoneum is observed; this is not seen in the control or shRNA-Par2-injected mice. Right panel: open abdomen. While disseminated ovarian cancer foci are seen in the ES2-inoculated mice, no cancer foci are seen in the shRNA-Par2-inoculated clones or in control non-injected mice (n = 7 mice per treatment). (**A**) Control non treated mouse. (**B**) sh RNA-Par2 inoculated ES2 cells into the peritoneum, clone1. (**C**) sh RNA-Par2 inoculated ES2 cells into the peritoneum, clone 2. (**D**) ES2 peritoneal inoculation, mouse 1. (**E**) ES2 peritoneal inoculation, mouse 2. (**F**) ES2 peritoneal inoculation, another view of mouse1.

When the i.p. injected mice showed a swollen abdomen (after 28 days), the experiment was terminated and then the peritoneum was examined. As demonstrated in Figure 3. wt ES2 injected mice exhibited multiple carcinoma foci disseminated throughout the peritoneum. In contrast, the inoculated shRNA-Par2 knockdown clones exhibited a relatively clean peritoneum, which was similar to the control non-injected mice (Figure 3).

### 3.4. PAR2-PH Binding Motif and Signaling Partners in Ovarian Cancer Cell Lines

**Figure 4:**
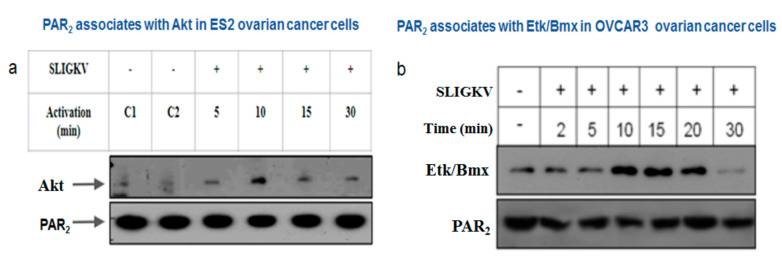
PAR2 forms a complex with PH-signal proteins in ES2 and OVCAR3 ovarian cancer cell lines. (**a**) ES2 ovarian carcinoma cells were activated with SLIGKV for 5′, 15′, and 30′. Application of anti-PAR2 abs was carried out on ES2 cell lysates to immunoprecipitate endogenous levels of PAR2. Akt was detected following Western blotting with anti-Akt abs. Increased association between PAR2 and Akt was observed 10 min after SLIGKV activation. (**b**) OVCAR3 cells were activated with SLIGKV for the indicated periods of time. Cell lysates were immunoprecipitated with anti-PAR2 Abs. Etk/Bmx was detected following Western blotting with anti-Etk/Bmx abs. Levels of Etk/Bmx are shown per equal PAR2 protein immunoprecipitated.

Akt/PKB is a serine/threonine protein kinase, which plays a pivotal role in tumor cell survival, proliferation, and invasiveness [29,30,31]. All three Akt/PKB isoforms include the conserved PH domain and a kinase domain near the N-terminal portion [32]. In order to elucidate events in the PAR2-PH binding motif-driven signaling network, we evaluated the PAR-AKT association in the ES2 ovarian cancer cell line, as was previously demonstrated in breast and colon cancers [10,11]. After SLIGKV PAR2 activation for different time periods, cell lysates were prepared followed by immunoprecipitation (IP) using anti-PAR2 abs. Western blot detection of Akt in the PAR2 immunoprecipitants was carried out by application of anti-Akt abs. We observed a temporary association of Akt with PAR2, maximally obtained endogenously after 10 min of SLIGKV activation, which declined thereafter (Figure 4a). This association was also demonstrated in HEK293 cells following ectopic transfection of YFP-Par2 and Akt whereby we have shown that the Akt-PH domain alone is associated with PAR2 while its mutant AKT PH-R25C fails to associate [9]. Hence, regardless of either endogenous PAR2 association in ES2 or following the overexpression of Par2 and Akt, the binding of Akt with PAR2 is observed.

The epithelial tyrosine kinase (Etk), also known as Bmx, is a non-receptor tyrosine kinase capable of associating with both GPCRs and tyrosine kinase receptors [32,33]. It mainly comprises a PH domain, one Src homology 3 (SH3)-, one SH2 site, and a tyrosine kinase domain [34,35,36,37]. We have previously characterized Etk/Bmx interactions with PAR1 as well as PAR2 [14,34]. For this, we evaluated the binding of cell lysates exhibiting various Par1 constructs to GST-Etk/Bmx. While Y397Z Par1 and wt Par1 associated with Etk/Bmx, lysates of truncated Par1 (devoid of the cytoplasmic tail) or JAR cells (lacking PAR1) showed no binding between PAR1 and Etk/Bmx [38]. Similarly, we previously showed that IP studies revealed an association between PAR2 and Etk/Bmx, 15–20 min following SLIGKV activation, which declined thereafter. In contrast, no binding was observed when a truncated form of Par2 lacking the entire cytoplasmic tail was used. This binding occurs via association of the PH domain of Etk/Bmx to the PAR2 C-tail, as we previously assessed using the GST-PH-Etk/Bmx pull-down assay [10]. Here, we show the endogenous association between Etk/Bmx and PAR2 in OVCAR3 cells. Maximal association is observed between 10 and 20 min following SLIGKV activation and declines thereafter (Figure 4b). We next assessed the impact of the P*c*(4-4) compound, which was selected out of a library and directed toward the PAR PH binding domain on the ovarian cancer mouse model, in vivo.

### 3.5. Characterization of PAR2-PH Binding Domain Peptides

**Figure 5:**
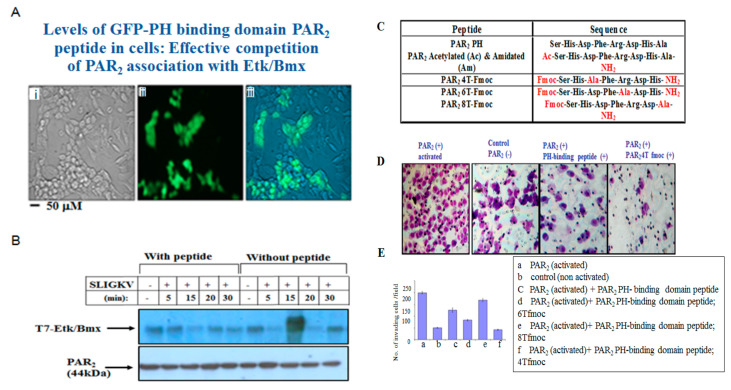
Characterization of PAR2-PH binding domain peptides. (**A**) GFP-PH binding domain peptide penetration to HEK293 cells (**ii**,**iii**) as compared to untreated cells (**i**). (**B**) Potent inhibition of the PAR2-Etk/Bmx association by the GFP-PAR2-PH binding domain peptide. HEK 293 cells transfected with Par2/f2rl1. Immunoprecipitation of cell lysates in the presence and absence of the peptide was performed, following SLIGKV activation for various time periods. Etk/Bmx was detected by anti-T7 abs. Levels of PAR2 immunoprecipitate were detected by anti-PAR2 abs. (**C**) Table showing the list of PAR2-PH binding domain peptides (wt and modified peptides). (**D**) Matrigel invasion in the presence and absence of PAR2 SLIGKV activation. Representative modified peptides are shown. (**E**) Histograms of the wt and various modified PAR2-PH binding domain peptides in the Matrigel invasion assay.

Targeting the PH binding motif of PAR2 can serve as a platform for potent inhibition of PAR2-induced ovarian cancer. A peptide representing the PAR2-PH binding motif was prepared and attached to GFP. This GFP–peptide is capable of effectively penetrating cells (Figure 5A). A simple chemical modification of the N-terminal peptide and the carboxy terminal heptapeptide of the PAR2-PH binding domain in combination with the “Alanine scan” approach was implemented in order to prepare a novel PAR2 peptide library based on the active heptapeptide SHDFRDH (Figure 5C). When the GFP-modified peptide was incubated with cells, abundant green fluorescence was seen within these cells, indicative of GFP–peptide penetration (Figure 5A, panels ii,iii) as compared to none in control cells without incubation with the GFP–peptide (Figure 5A, panel i). In the presence of the GFP-PAR2-PH binding domain peptide, a marked inhibition of PAR2-Etk/Bmx complex formation was seen. In-contrast, in the absence of this GFP–peptide, an association between PAR2-Akt was seen, following SLIGKV activation (Figure 5B). In an effort to determine the impact of chemical modifications and the significance of each specific residue to peptide activity, a mini library of the chemically modified peptide was generated, as outlined in the table in Figure 5C. The activity was evaluated via Matrigel invasion assay of wt PAR2 before and after SLIGKV PAR2 activation compared to the modified peptides. A representative Matrigel invasion assay is shown (Figure 5D). In this model, the peptide library demonstrated structure-related activity. Acetylated and amidated PAR2 peptide resulted in diminished Matrigel invasion activity. Arginine at position 5 is also important for the activity of the peptide and its substitution results in a diminished invasive activity (6T-Fmoc). Substituting the His at position 7 disables the inhibition of PAR2 invasion activity through Matrigel (the peptide named 8T-Fmoc, shown as a histogram). Peptide 4T-Fmoc shows that aspartic acid in position 3 is not important for the peptide activity, and, therefore, peptide 4T Fmoc effectively inhibited Matrigel invasion of PAR2-activated ES2 cells (Figure 5D).

### 3.6. Pc(4-4) Inhibits PAR2-Akt Association and Ovarian Cancer Peritoneal Dissemination

**Figure 6:**
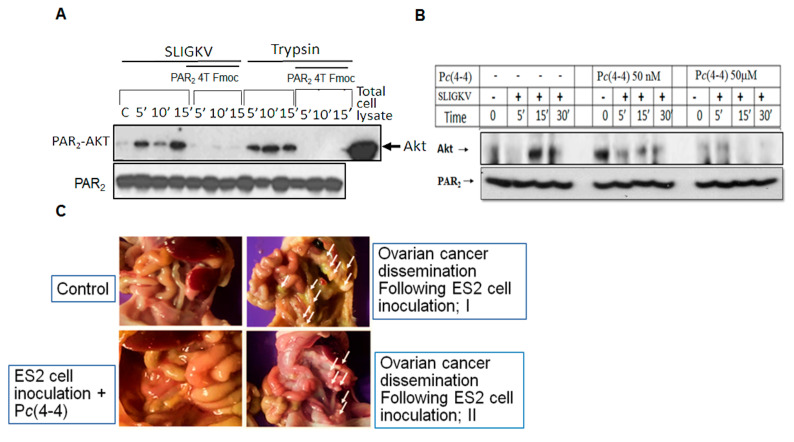
P*c*(4-4), the PAR2-PH binding domain-directed peptide, inhibits the PAR2-Akt association. (**A**) HEK293 cells were transiently transfected with Par2 followed by SLIGKV or trypsin PAR2 activation in the presence and absence of PAR2 4T Fmoc P*c*(4-4). IP of cell lysates following different periods of time for activation was carried out using anti-PAR2 abs. Detection of Akt was performed using anti-Akt abs. (**B**) P*c*(4-4) compound inhibits PAR2-Akt association. HEK293 cells were transiently transfected with Par2 followed by SLIGKV PAR2 activation in the presence and absence of 50 μM P*c*(4-4). IP of cell lysates following different durations of activation was carried out using anti-PAR2 abs. Detection of Akt was performed using anti-Akt abs. β-actin serves as a control gene for loading. (**C**) P*c*(4-4) inhibits ovarian cancer growth and peritoneal dissemination. Mice were injected i.p. with wt ES2 cells (1 × 10^6^) and were treated or not with P*c*(4-4) compound directed toward PAR PH binding domain (5 mg/kg). After 30 days, mice were terminated, and pictures of the open abdomen are shown. While distributed ovarian cancer foci are seen in the abdomen of the ES2-inoculated mice, almost no cancer foci are seen in the P*c*(4-4)-treated mice (n = 7 mice per treatment).

HEK293 cells were transiently transfected with Par2/f2rl1 and IP analysis was performed. In the presence of a modified PAR2-PH binding domain peptide; PAR2 4TFmoc (50 μg/mL), a potent inhibition of the PAR2-Akt association, is observed. This is seen regardless of whether it is activated via SLIGKV or trypsin (Figure 6A). Upon application of P*c*(4-4), a lead cyclic peptide directed to the PAR2-PH binding domain prior to the IP analysis, a partial inhibition was seen at 50 nM as compared to strong inhibition of Akt/PKB-PAR2 association at 50 μM (Figure 6B) following either SLIGKV or trypsin activation of PAR2. Having demonstrated the inhibitory effect of P*c*(4-4) in preventing the association between Akt/PKB and PAR2, we next set out to evaluate its effect on ovarian cancer in vivo.

The physiological significance of the PAR2-PH binding motif on ovarian cancer in vivo is demonstrated using a xenograft mouse model of ES2 cells inoculated i.p. in the presence and absence of P*c*(4-4). P*c*(4-4) cyclic peptide was applied repeatedly three times/week (5 mg/kg). ES2 cell inoculation generated multiple ovarian cancer foci, which spread and disseminated in the peritoneal compartment. The experiment was terminated after 32 days. After that time, the abdomen of mice that were treated with P*c*(4-4) revealed almost no ovarian cancer foci and the surrounding abdomen was clear (Figure 6). In contrast, abundant disseminated ovarian cancer foci were observed in the untreated mice. Hence, knockdown of Par2 (Figure 3A) or blocking of PAR2-mediated cell signaling via the P*c*(4-4)compound (Figure 6C) both resulted in significant inhibition of ovarian cancer growth and peritoneal dissemination.

## 4. Discussion

In this communication, we show the central role played by Par2/f2rl1 as a predictor of ovarian cancer. Par2/f2rl1 is highly expressed in the FT tissues of BRCA carriers as opposed to no expression in normal FT tissues. The fact that PAR2 is overexpressed in high-risk BRCA FTs may serve as a predictive indicator for ovarian cancer development. We demonstrate the significant role of PAR-PH binding site governing the molecular mechanism of PAR2-induced ovarian cancer. The association of either PH-Akt or PH-Etk/Bmx with the PAR2-PH binding motif in ovarian cancer cells provides a mechanistic rationale for the potent inhibitory effect observed by P*c*(4-4), our lead cyclic peptide, toward PAR2-PH binding domain and in ovarian cancer growth dissemination. For this purpose, we utilized web server analyses, and Par2/f2rl1 knockdown showing inhibition of ovarian cancer and peritoneal spread in mice, in vivo. Our novel P*c*(4-4) compound directed toward the PAR2-PH binding motif may serve as a therapeutic strategy in STICs and ovarian cancer.

The tumor microenvironment differs from that of the surrounding non-cancerous tissues, creating a milieu of gene signatures favoring tumor development. A search of the ovarian cancer microenvironment revealed the presence of abundant serine proteases present in either immobilized or soluble forms, as evidenced from a gene signature deduced by a microarray-based tumor microenvironment oncobiome [39]. Among these are trypsin/trypsinogen- [27], TF-VIIa- [40], matriptase- [41], and kallikrein (KLK)-related peptidases [42], all of which can potently activate PARs and most likely PAR2. Their overexpression at sites of the tumor microenvironment provides a molecular link for PAR2 functional activation in ovarian cancer. Indeed, we demonstrate that when Par2/f2rl1 is knocked down, the peritoneal dissemination and formation of ovarian cancer foci are effectively inhibited.

The evolving paradigm of FTs as the origin of HGSC has provided the basis to investigate the initial molecular changes occurring in FT tissues. In addition to p53 somatic mutations and BRCA1/2 genes that are engaged in the repair of double strand DNA breaks, other molecular mechanisms may be involved. For example, spatial transcriptomic analysis between serous tubal intraepithelial carcinoma (STICs), and their normal matched FTs indicated, among other changes, increased protein levels of insulin-like growth factor binding protein-2 (IGFBP2) [43]. This was shown to be due to epigenetic changes in the DNA methylation status. Hypomethylation in the IGFBP2 gene upstream sequence led to increased levels of the protein, independent of estrogen (E2). Another pathway of IGFBP2 expression levels, dependent on E2, has been shown to be limited to proliferative secretory cells. E2 takes a major role in the functioning of the female reproductive organs. Indeed, examination of pre- and postmenopausal FT tissues expressing or lacking E2, respectively, has indicated that in postmenopausal FTs, the levels of IGFBP2 are markedly reduced as opposed to premenopausal FTs [42]. While we have previously demonstrated E2 regulation of PAR1/f2r [44], it is most likely that E2 also regulates the expression of PAR2/f2rl1. Upstream PAR2/f2rl1 gene sequence analysis points to the presence of multiple estrogen response elements (ERE) (preliminary data), suggesting the likely regulation of PAR2/f2rl1 by E2. It has been proposed that in postmenopausal FTs where most HGSC cases occur, an alternative pathway arises, independent of E2, which utilizes an epigenetic mechanism of IGFBP2 gene promoter methylation [43].

Ovarian cancer seeding from a primary tumor in the FTs, which already contain driver gene alterations, participates in the process that leads to malignant ovarian cancer. Whole-exome sequencing and copy number analyses of laser capture micro-dissected FT lesions as well as ovarian tumors demonstrated the involvement of TP53, the PI3K pathway, PTEN, and BRCA1/BRCA2 as driver genes [22]. Additional proteins that are overexpressed in STICs have been proposed. RNA sequencing comparing transcriptomes between HGSC and normal FT epithelial tissues highlighted LAMC1 gene encoding for the laminin γ1 chain, as expressed in STICs but not in normal tissues. Laminin is a basement membrane protein and its overexpression in STICs may well contribute to the attachment and spread of STIC cells to the peritoneum via interactions with its α3β1 integrin receptor, abundantly present on the surface of the peritoneal mesothelium [45].

Rsf-1, also known as HBXAP, was found to be upregulated in STICs [46]. Rsf-1 is a histone chaperone mediating ATP-dependent chromatin remodeling, which is necessary and essential for transcriptional activation (or repression) as well as for cell cycle progression and DNA replication, which are typical of cancer pathogenesis. Importantly, HGCS patients overexpressing Rsf-1 have a shorter overall survival time compared to HGCS patients that do not exhibit upregulated Rsf-1. In parallel, through this line of evidence, Cyclin E, encoded by CCNE1, is an established cell cycle protein, forming a complex with Cdk2 and promoting the shift from the G1 to S phase of the cell cycle, a function required for cancer malignancy [46]. Stathmin 1 (STMN1), an oncogene involved in the regulation of cell motility and cell division via controlling microtubule dynamics, is also overexpressed in STICs and HGSCs [47]. STMN1 contributes most likely to tumor spread and dissemination. The current study demonstrating that PARs are overexpressed in the FTs of BRCA1/2 patients as opposed to being absent in normal FT tissues could be strengthened and could benefit from similar evaluations in the genetically engineered mouse model of HGSC using the PAX8 transcription factor to target FTs [26].

The involvement of PAR2 in ovarian cancer, has been previously demonstrated [27,28,29,48]. PAR2 was shown to be significantly overexpressed in clinical tissues of ovarian cancer via association with β-arrestin and cross-talk with epidermal growth factor receptor (EGFR) [28]. In addition, trypsin, the physiological ligand of PAR2, was shown to be stabilized in the presence of HE4, a WAP-family glycoprotein, that inhibits trypsin degradation and was found to be increased in ovarian cancer [27]. Recently, levels of matriptase, a transmembrane serine protease, were shown elevated in ovarian cancer, acting via the activation of PAR2 inducing the signaling of PI3K/Akt and MMP9 promoted the dislodging of cell–cell contacts [29].

Among the challenges ahead and future studies are the identification of the entire landscape of TF gene profiles for early predictors of ovarian cancer and as effective targets for therapy. Recently we have assigned RNF43 as a negative regulator of PAR2 similar to its action on FZDs [17]. Inactivating RNF43 mutations in blood samples of ovarian cancer patients were detected [15], leading consequently to high Wnt signaling and stabilization of β-catenin. It is important to evaluate the impact of these RNF43 mutations on levels of PAR2 in ovarian cancer. This is significant for the purpose of detecting the best responding ovarian cancer patient population to PAR-involved therapy.

Here, we demonstrate the abundant IHC staining of PAR2 in BRCA carriers FTs as opposed to no staining in normal FT tissues. A well-designed genetic mouse model presented by the Drapkin lab [26] demonstrated unequivocally that HGSC originates in the fallopian tubal secretory epithelial cells, which recapitulate the main genetic modifications and lesions typical of human invasive ovarian cancer. We propose that PAR2 joins the known high-risk indicators and may serve as a potent detection sensor for ovarian cancer.

We have selected a lead cyclic peptide P*c*(4-4) from a library of cyclic peptides, directed toward the PH binding motif. We found that P*c*(4-4) is a powerful inhibitor of PAR4- and PAR2-PH-associated signal proteins [11]. As such, it is shown here that P*c*(4-4) effectively inhibits ovarian cancer development and peritoneal dissemination in vivo, in a mouse xenograft ovarian model. Together with this line of evidence, our unpublished data show that P*c*(4-4) acts via stabilization of the wt p53 tumor suppressor gene and inhibition of Mdm2, a p53 ubiquitin ligase, as well as c-Myc oncogene levels.

Cyclic peptides often imitate a protein secondary structure representing prolonged stability in an enzyme-enriched environment. Backbone cyclization mostly employs atypical building blocks with a linker of customizable length covalently attached to a backbone active group for cyclization. Optimization of the PAR-PH binding domain peptide cyclization, in terms of the ring size, results in enhanced stability, and the P*c*(4-4) displays longstanding effects on tumor development in mice as we previously demonstrated [11] and showed here. However, for human therapy, it will still have to undergo careful oral bioavailability evaluations and absorption efficiency tests.

The cross-talk between receptor tyrosine kinases (RTK), EGFR/erbB, and GPCRs, including PARs, is now well accepted [49,50,51]. We have recently demonstrated active cross-talk between PAR4 and EGFR/erbB [11], as was also demonstrated for PAR2-EGFR by others [28]. It was shown that activation of PAR2 instigated Gαq/11, Gα12/13, and β-arrestin1/2, leading to downstream src kinase activity and the transactivation of EGFR. We propose a direct link between PARs and EGFR via the association of PH-signal proteins, such as Etk/Bmx, Gab1, or Sos1 [11], capable of potently associating via the PAR2 or PAR4 PH binding motifs at their C-tails. As such, Sos1 may serve as a junction protein mediating the direct cross-talk between PARs and EGFR/erbB. Association of Sos1 with Grb2 recruits Shc that binds to Tyr phosphorylated erbB EGFR family members via its SH2 domain.

In conclusion, we hereby assign PAR2 as part of the gene signature which may serve as a predictor of ovarian cancer, highly expressed in the FTs of BRCA1/2 patients as opposed to there being none in normal tissues of FT. In parallel, we addressed the molecular mechanism of PAR2 stem cell in the etiology of ovarian cancer, demonstrating the significance of the PH binding motif of PAR2 critical for ovarian cancer growth dissemination. As such this motif serves as a potent platform for the design of a cyclic peptide library directed toward PAR2-PH binding motif. P*c*(4-4), a selected lead compound, exhibits “drug-like” properties in terms of metabolic stability, solubility, clearance, and toxicity, as we have previously described [11]. The effective inhibitory impact of P*c*(4-4) is demonstrated via abrogation of PAR2-PH-Akt association, in vitro, and ovarian cancer peritoneal growth dissemination, in vivo. Overall, this indicates P*c*(4-4) as a therapy pilot in ovarian cancer.

## Data Availability

Data that support the findings of this study are available upon request by the corresponding author.
